# Procurement of heart and heart-lungs block with simultaneous abdominal normothermic regional perfusion

**DOI:** 10.1016/j.xjtc.2024.02.025

**Published:** 2024-03-11

**Authors:** Prashant N. Mohite, Simon Messer, Philip Curry

**Affiliations:** Department of Cardiac Surgery and Transplantation, Golden Jubilee National Hospital, Glasgow, United Kingdom

**Figure:**
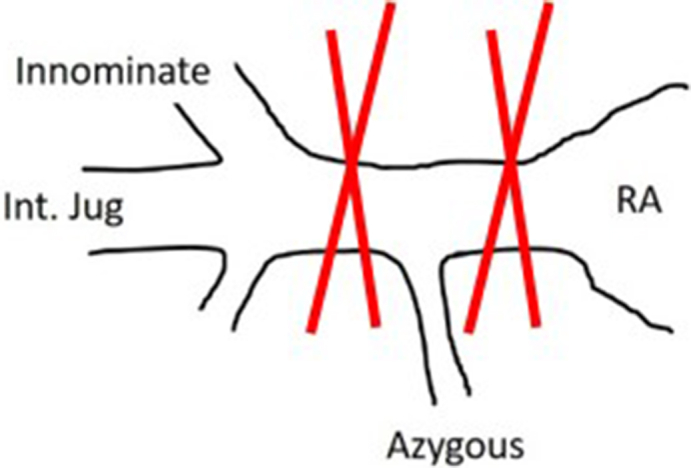
Isolation of azygous vein in an emergency setting.

**Central Message**

A priority-based stepwise approach helps conserve blood and avoid blood loss in the procurement of donor heart and lungs in a DCD situation in combination with abdominal NRP.

Abdominal normothermic regional perfusion (A-NRP) is increasingly used during donation after circulatory death (DCD) due to encouraging outcomes in liver transplantation.^1^ However, simultaneous retrieval of the donor heart and lungs poses a challenge due to bleeding in the chest. It causes loss of volume in the A-NRP circuit leading to unstable flow and potentially compromising the perfusion of abdominal organs. Time spent controlling bleeding in the chest may delay procurement of thoracic organs leading to increased warm ischemia time, a chance of declining the organs, and higher incidence of primary graft failure. A stepwise approach based on the priority of organ preservation helps conserve blood and avoid blood loss (See Figure 1). Following circulatory death and 5 minutes of no-touch period, donor organ procurement is begun.

**Figure 1:**
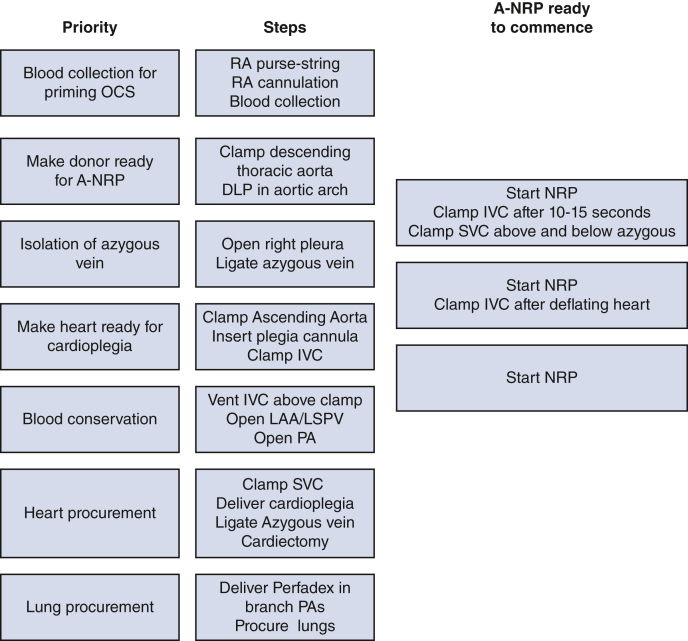
Priority-based stepwise approach. *OCS*, Organ Care System; *RA*, right atrium; A-*NRP*, abdominal normothermic regional perfusion; *IVC*, inferior vena cava; *SVC*, superior vena cava; *LAA*, left atrial appendage; *LSPV*, left suprior pulmonary vein; *PA*, pulmonary artery.

## Heart-Only Retrieval

### Priority 1

Donor hearts retrieved after DCD are assessed with the ex vivo platform, that is, Organ Care System (OCS), which requires priming with approximately 1.2 L donor blood. It is drained into a blood collection reservoir through a cannula introduced into the right atrium (RA). The cannula is put through a purse string that is tied after blood collection to prevent blood loss after removal of the cannula. The absence of coronary artery disease is established before blood collection to avoid blood loss in case the heart is not suitable for transplantation.

### Priority 2

It is essential is to make the donor NRP ready by clamping the descending thoracic aorta and introducing a vent (DLP cannula) into the arch to ensure a lack of cerebral perfusion.

### Priority 3

Isolation of the azygous vein is carried out by opening the right pleura and accessing the vein superior to the right hilum. It is either ligated, stapled, or liga-clipped.

### Priority 4

Cardioplegia resuscitates an arrested heart and it is an utmost priority after above steps. In preparation of cardioplegia, the ascending aorta is clamped proximal to the arch-venting-DLP and a cardioplegia cannula is introduced proximal to this clamp. IVC is clamped above the diaphragm so that cardioplegia does not contaminate the A-NRP.

### Priority 5

However, before cardioplegia delivery, it is crucial to collect blood from the chambers of heart and lungs. It is done by venting the IVC between the clamp and the RA that drains the right heart and supradiaphragmatic body, venting the left superior pulmonary vein that drains blood in the postcapillary pulmonary circulation, and venting the left pulmonary artery that drains blood from the precapillary pulmonary circulation.

Blood is collected into the blood collection reservoir mentioned above. An amount of 1.2 L blood is used for the OCS priming while the rest is returned to the A-NRP reservoir as required. Once the blood is collected, cardioplegia is delivered along with topical cooling. The donor heart is procured by cutting the left atrium, pulmonary veins, IVC, superior vena cava, pulmonary artery, and the aorta. The pulmonary arteries and pulmonary veins can be individually staple-cut to prevent bleeding following cardiectomy.

While performing above steps, the A-NRP can be commenced anytime after clamping of the descending thoracic aorta and introducing DLP into the arch. As shown in Figure 1, if A-NRP is ready to start before cardioplegia, the IVC can be clamped 10-15 seconds after A-NRP is commenced to deflate the heart. However, if the A-NRP is not ready to start until Cardioplegia delivery, the IVC is required to clamp and the blood in the heart and upper part of the body is then colletced by venting IVC cephalad to the clamp.

**Figure 2:**
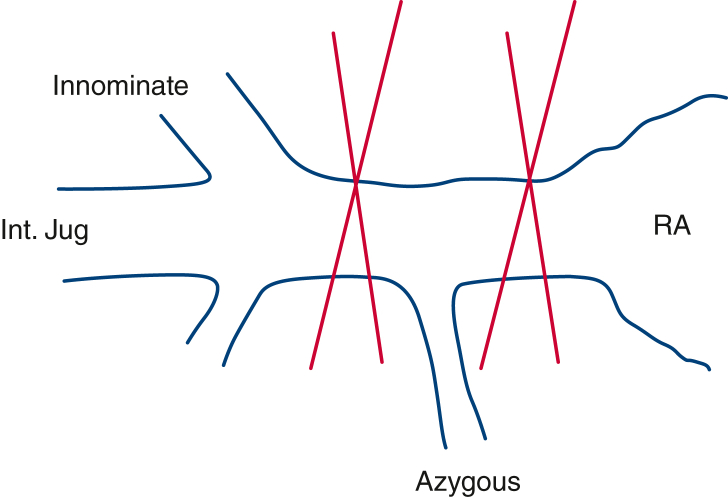
Isolation of azygous vein in an emergency setting. *Int. jug*, Internal jugular vein; *RA*, right atrium.

• **Tip 1:** Clamping of the IVC 10 to 15 seconds after commencement of the A-NRP allows drainage of the supradiaphragmatic body and right-sided chambers of the heart into the A-NRP reservoir. Therefore, the IVC clamping should be delayed until the abdominal team commences the A-NRP as long as it happens before the delivery of the cardioplegia. Cardioplegia should not be delayed for the A-NRP to start.
• **Tip 2:** If the azygous vein could not be ligated before commencement of the A-NRP, it is isolated by clamping the superior vena cava at 2 places: near its junction to the RA and near its junction with the innominate vein (Figure 2). It avoids delay in commencing A-NRP and cardioplegia delivery. The azygous vein can be later dissected and ligated at the time of cardiectomy.

## Heart and Lung Retrieval

Initial steps of blood collection for the OCS priming and clamping of major vessels in the chest remain similar to the heart-only retrieval; however, with lungs being procured for transplantation the blood is vented through left atrial appendage and the distal main pulmonary artery trunk. Following cardiectomy, the antegrade pulmonary perfusion is carried out before lung procurement.

## Discussion

**Figure E1:**
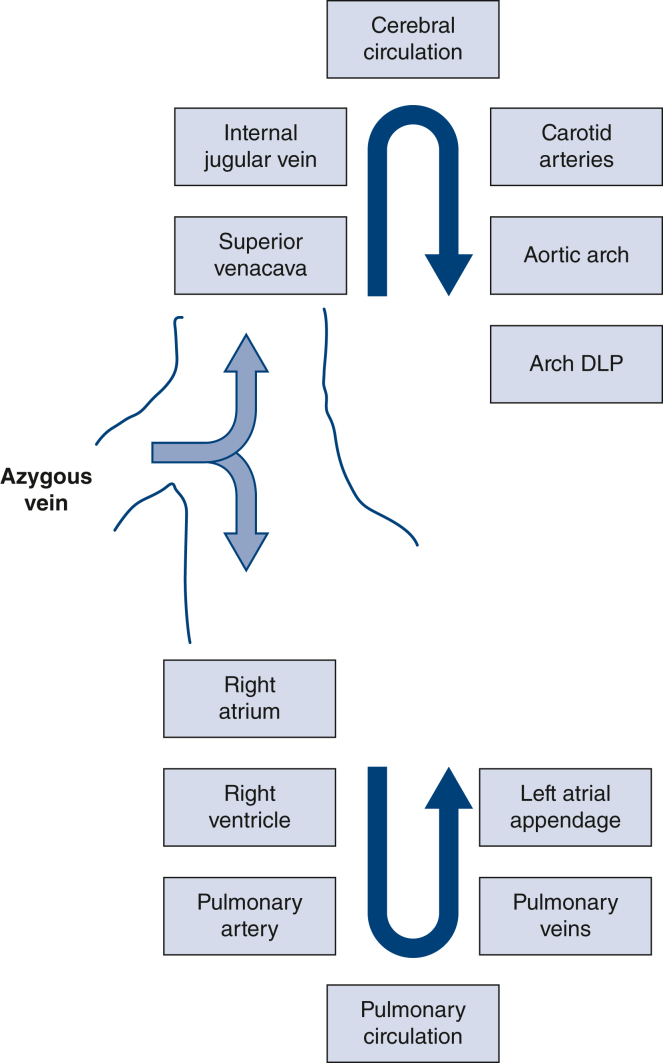
Bleeding due to inadequate isolation of the azygous vein.

Previously, we have described the world's first direct procurement of donor heart along with A-NRP.^2^ In the present article, we present our refined technique with a focus on blood conservation. Institutional review board approval and patient consent were not required. The azygous vein, if not isolated, may drain a significant volume of blood from A-NRP through the RA and internal jugular vein as shown in Figure E1. The possibility of donor brain perfusion cannot be ruled out in cases of inadequate isolation of the azygous vein and the superior vena cava. Because facilitation of A-NRP and the delivery of cardioplegia remains the priority, there may not be sufficient time to dissect and ligate the azygous vein. We recommend isolating the azygous vein by clamping the superior vena cava at its junction to the RA and near its junction with the innominate vein to facilitate the A-NRP and cardioplegia delivery.

Right side chambers of the heart and precapillary and postcapillary parts of the lung vasculature must be drained by opening the RA, pulmonary artery, and the left atrial appendage, respectively. This blood is used in the NRP reservoir to maintain its circulatory volume and flow.

## Conflict of Interest Statement

The authors reported no conflicts of interest.

The *Journal* policy requires editors and reviewers to disclose conflicts of interest and to decline handling manuscripts for which they may have a conflict of interest. The editors and reviewers of this article have no conflicts of interest.

## References

[1] Watson C.J.E., Hunt F., Messer S. (2019). In situ normothermic perfusion of livers in controlled circulatory death donation may prevent ischemic cholangiopathy and improve graft survival. Am J Transplant.

[2] Mohite P.N., García Sáez D., Butler A.J. (2019). Direct procurement of donor heart with normothermic regional perfusion of abdominal organs. Ann Thorac Surg.

